# Protocol for SARS-CoV-2 post-vaccine surveillance study in Australian adults and children with cancer: an observational study of safety and serological and immunological response to SARS-CoV-2 vaccination (SerOzNET)

**DOI:** 10.1186/s12879-021-07019-1

**Published:** 2022-01-20

**Authors:** Amy Body, Elizabeth Ahern, Luxi Lal, Karen Gillett, Hesham Abdulla, Stephen Opat, Tracey O’Brien, Peter Downie, Stuart Turville, C. Mee Ling Munier, Corey Smith, C. Raina MacIntyre, Eva Segelov

**Affiliations:** 1 Level 7, Monash Health Translational Precinct, 246 Clayton Rd, Clayton, Melbourne, VIC 3168 Australia; 2 Monash University, Clayton, Melbourne, VIC 3168 Australia; 3 Kids Cancer Centre, Sydney Children’s Hospital’, Randwick, NSW 2031 Australia; 4 School of Women’s & Children’s Health, Faculty of Medicine, University of New South Wales, Sydney, 2052 Australia; 5 Children’s Cancer Centre, Monash Children’s Hospital, 246 Clayton Rd, Clayton, Melbourne, VIC 3168 Australia; 6 Immunovirology and Pathogenesis Program, The Kirby Institute, University of New South Wales, Kensington, Sydney NSW 2052 Australia; 7 QIMR Berghofer Centre for Immunotherapy and Vaccine Development and Translational and Human Immunology Laboratory, QIMR Berghofer Medical Research Institute, Brisbane, QLD Australia; 8 The Kirby Institute, University of New South Wales, Kensington, Sydney NSW 2052 Australia

**Keywords:** SARS-CoV-2 vaccine, Covid-19 vaccine, Cancer, Vaccine response, Immune response

## Abstract

**Background:**

Cancer is associated with excess morbidity and mortality from coronavirus disease 2019 (COVID-19) following infection by the novel pandemic coronavirus SARS-CoV-2. Vaccinations against SARS-CoV-2 have been rapidly developed and proved highly effective in reducing the incidence of severe COVID-19 in clinical trials of healthy populations. However, patients with cancer were excluded from pivotal clinical trials. Early data suggest that vaccine response is less robust in patients with immunosuppressive conditions or treatments, while toxicity and acceptability of COVID-19 vaccines in the cancer population is unknown. Unanswered questions remain about the impact of various cancer characteristics (such as treatment modality and degree of immunosuppression) on serological response to and safety of COVID-19 vaccinations. Furthermore, as the virus and disease manifestations evolve, ongoing data is required to address the impact of new variants.

**Methods:**

SerOzNET is a prospective observational study of adults and children with cancer undergoing routine SARS-CoV-2 vaccination in Australia. Peripheral blood will be collected and processed at five timepoints (one pre-vaccination and four post-vaccination) for analysis of serologic responses to vaccine and exploration of T-cell immune correlates. Cohorts include: solid organ cancer (SOC) or haematological malignancy (HM) patients currently receiving (1) chemotherapy, (2) immune checkpoint inhibitors (3) hormonal or targeted therapy; (4) patients who completed chemotherapy within 6–12 months of vaccination; (5) HM patients with conditions associated with hypogammaglobulinaemia or immunocompromise; (6) SOC or HM patients with allergy to PEG or polysorbate 80. Data from healthy controls already enrolled on several parallel studies with comparable time points will be used for comparison. For children, patients with current or prior cancer who have not received recent systemic therapy will act as controls. Standardised scales for quality-of-life assessment, patient-reported toxicity and vaccine hesitancy will be obtained.

**Discussion:**

The SerOzNET study was commenced in June 2021 to prospectively study immune correlates of vaccination in specific cancer cohorts. The high proportion of the Australian population naïve to COVID-19 infection and vaccination at study commencement has allowed a unique window of opportunity to study vaccine-related immunity. Quality of life and patient-reported adverse events have not yet been reported in detail post-vaccination for cancer patients.

*Trial registration* This trial is registered on the Australia New Zealand Clinical Trials Registry (ANZCTR) ACTRN12621001004853. Submitted for registration 25 June 2021. Registered 30 July 2021 (Retrospectively registered). https://www.anzctr.org.au/Trial/Registration/TrialReview.aspx?id=382281&isReview=true

**Supplementary Information:**

The online version contains supplementary material available at 10.1186/s12879-021-07019-1.

## Background

More than 18 months into the COVID-19 pandemic, major social and economic disruption has occurred worldwide. To date, more than 4.7 million people globally have died from COVID-19 disease and more than 230 million have been infected [1].

### Impact of the COVID-19 pandemic on people living with cancer

The COVID-19 pandemic has had significant impacts on the lives of people living with cancer. Treatment and follow up have been impacted by disruptions in normal hospital activities, with resources diverted to managing COVID-19 outbreaks, and intentional rationalisation of hospital visits and treatment to minimise risk of COVID-19 exposure for patients [2].

Furthermore, there has been a major impact on usual screening procedures and pathology notifications during the pandemic [3]. A 10% lower rate of pathology notifications from April until October 2020 than that expected during the same time period was noted in Victoria, Australia [4]. More marked decreases in notifications were noted in high COVID-19 incidence countries such as the United States, with 56.9% fewer cases of cancer notified in April 2020 in the US than expected [5]. It is anticipated that in the coming years there will be a corresponding increase in diagnoses of cancers at a later stage due to these missed opportunities for early diagnosis [6]. An increase in both number and acuity of diagnoses of cancer will result in high numbers of patients undergoing active cancer treatment in the coming years.

COVID-19 infection has more severe outcomes for patients with cancer, with excess mortality noted in cohorts around the world [7–9]. Cancer is a heterogeneous disease representing a wide range of patients. The outcome of COVID-19 amongst cancer patients differs according to the primary site of the tumour and between those with localised versus metastatic disease [7, 10]. Cancer as an entity may not fully explain the poor outcomes seen in many patients—a European study found that controlling for age and comorbidity resolved the difference in COVID-19 outcome seen between cancer and non-cancer patients [11]. Notwithstanding this, a systematic review and meta-analysis found a risk of 30-day mortality of COVID-19 infection in cancer patients to be 30% in hospitalised patients, and 15% in cohorts with mixed inpatient and outpatient groups [12]. Age was also a risk factor for death in this study, but cancer-related factors (haematological malignancies, recent treatment) remained independent predictors of mortality, suggesting that not all deaths are explained by age and comorbidity. The cancer population is, therefore, a vulnerable group due to age and comorbidity, but also with additional risk factors including active treatment and, in some cases, the cancer itself.

Increased risk of severe COVID-19 outcomes and death from COVID-19 disease has also been noted in the paediatric cancer setting. An international registry study of 1500 children with cancer and COVID-19 found that 3.8% of infected patients died [13], compared with a risk of death from COVID-19 of 0.005% in the general paediatric population [14, 15].

COVID-19 infection following immunosuppressive treatments can also lead to chronic infection of greater than 5 months duration during which time there is persistent and accelerated evolution of the virus, with potential implications for public health related to the development of new variants [16].

### Response to vaccination in cancer patients

Protection against COVID-19 afforded by vaccination relies on an adequate immune response. Despite excellent protection against severe COVID-19 in the general population, there is concern that the vaccines will not be as effective in immunocompromised patients.

#### Impact on the clinical presentation of COVID-19 disease

Data regarding outcomes of COVID-19 infection after vaccination in patients undergoing cancer treatment are lacking although data from other immunosuppressed patient populations suggest an ongoing risk of severe COVID-19. A report from the solid organ transplant (SOT) population reported a high rate of hospitalisation (27%), intensive care admission (11%) and death (5%) in 55 solid organ transplant recipients who had become infected with COVID-19 after vaccination, suggesting that the degree of protection afforded against clinical illness in immunocompromised populations may be lower [17]. Concordantly, prevalence of anti-SARS-Cov-2 antibodies after two doses of BNT162b2 vaccine in 101 French SOT patients was only 40% [18].

A predictive model for risk of hospital admission or death from COVID-19 after vaccination found that recent chemotherapy and diagnosis of a blood cancer were among the list of risk factors for poor outcome despite vaccination [19].

#### Serological response

In the cancer setting, 2 large prospective trials have been presented at the European Society of Medical Oncology (ESMO) congress (September 2021) [20, 21]. These studies found high seroconversion rates to SARS-CoV-2 vaccination in adult patients with cancer (The VOICE study, 83–93% depending on arm [20], the CAPTURE study, 78% [21]). The CAPTURE study included haematological malignancies and found lower rates of seroconversion in this group. Lower rates of neutralising antibodies were noted to variants of concern. Both studies have so far only reported follow up until 2–4 weeks post second vaccine dose. Previously published studies have focused on short term serologic and immunologic response to vaccination in relatively small cohorts with diverse patient characteristics. Early evidence suggests a possible influence of treatment modality and cancer type upon antibody response, with impairment noted in those with haematological malignancies or receiving myelosuppressive treatments such as chemotherapy. In addition, these data confirm the importance of a second (boost) vaccine dose in cancer patients as critical to achieve improved antibody response to the vaccine. Summarised data is included in Table 1.

**Table 1:** Summary of seroconversion rates and T cell responses amongst patients with cancer and healthy adults

	Seroconversion post 1 dose	Seroconversion post 2 doses	T cell response	Modifying factors
Solid organ cancer patients	38–83% [22, 24, 49, 50]	84–98% [20, 21, 24, 25, 49]	71% [24]	Use of steroids [24] Combined chemoimmunotherapy [23, 24]
Haematological malignancies	18–72% [24, 49, 51]	60–85% [20, 21, 24, 25, 27, 49]	50% [24]	Anti CD20 treatment [25] Stem cell transplant [25] BCMA targeted therapy [27] Anti CD38 regimens [27] Disease response [51]
Healthy adults	100% [28, 52]	100% [28, 52]	94% [28]	Age [28]

Detectable anti-Spike antibodies

*BCMA* B cell maturation antigen

Despite relatively preserved rates of seroconversion following the second vaccine dose in patients with SOC, there additionally remain concerns about lower titres of protective antibodies in patients with cancer. Several investigators have found lower titres of neutralising antibodies in SOC patients [22, 23]. Others have found that in SOC patients who seroconvert, titres are similar to healthy controls [24, 25]. With regard to haematological malignancies, these patients consistently demonstrate lower antibody titres than healthy controls [25–27].

This area requires further research to elucidate the clinical predictors of reduced antibody titres and the consequence of lower titres for protection against symptomatic COVID-19.

#### Cellular immunity

The importance of T-cell-mediated immunity to optimal vaccine responses is increasingly recognised, and T-cell correlates of vaccine efficacy [including type-1 cytokine production by peripheral T cells such as interferon-gamma (IFN-γ)] have been demonstrated in early-phase trials of leading COVID-19 vaccines [28, 29]. Impaired peripheral T-cell immunological status in cancer patients is a further concern, with documented reductions in circulating T cell subsets and differences in T cell phenotypes demonstrated in this group compared with healthy controls [30]. Interestingly, in small subsets of patients Monin et al. demonstrated no clear deficiency of SARS-CoV-2-specific T-cell responses to receptor binding domain or spike antigen following prime vaccine in SOC patients compared with healthy controls although significantly worse responses were seen in a small cohort of haematological malignancy (HM) patients [24].

#### Serologic and immunologic response to SARS-CoV-2 vaccination in children with cancer

To date, there are no published data regarding the outcome of COVID-19 vaccination for children with cancer. In the healthy clinical trial population who underwent vaccination with the BNT162b2 vaccine, neutralising antibody titres in children aged 12–15 years were higher than seen in young adults aged 16–25 receiving the same vaccine [31]. It is unknown how childhood cancer treatments (many of which induce significant myelosuppression) may impact on vaccine outcome.

### Safety of vaccination in cancer patients

Published data have not shown any increase in toxicity in cancer patients compared with healthy controls. In fact, Monin et al. showed reduced toxicity rates in patients, potentially related to the lower immunogenicity of the vaccines in this cohort [24]. There has been concern regarding potential triggering of immune-related adverse effects (iRAE) by vaccination in patients on immune checkpoint inhibitors, however a short-term study in Israel of 137 patients on immune checkpoint inhibitors showed no increase in irAE during follow up (until a median of 19 days post the second dose) [32]. Longer term safety data have not been published in cancer patients. Of particular concern in the cancer community is the known small risk of thrombotic events with the ChAdOx1-S vaccine, which appears to be antibody mediated [33]. Although the mechanism appears to be distinct from that of cancer-associated thrombosis, many patients and practitioners are worried about the potential for an increased risk of this complication in patients already at risk of thrombosis.

There are some potential safety concerns with vaccination in patients sensitised to polyethylene glycol (PEG, present in BNT162b2 vaccine) and polysorbate 80 (present in ChAdOx1-S). These excipients are found in many common cancer treatments and cancer patients sensitised to these agents with prior allergic reactions may be at higher risk of vaccine-related adverse effects [34, 35].

There is to date no published data on quality of life related to vaccination, which could potentially deteriorate due to adverse effects or improve due to less requirement for social isolation.

### The Australian COVID-19 experience

Since the onset of the COVID-19 pandemic in early 2020, Australia has imposed strict suppression measures to eliminate community transmission of COVID-19 [36]. As a result, the majority of Australians have not been infected with COVID-19. At commencement of study enrolment in June 2021; Australia, in initially pursuing a COVID-19 elimination strategy [37], had restricted confirmed COVID-19 infections to < 0.2% of the population [38]. However, vaccination rollout in Australia was relatively slow: at July 17 2021, Australia had the lowest share in the OECD of the population fully vaccinated at 10.8% [39]. This has allowed the SerOzNET study to capture a large cohort of unvaccinated participants who are also unexposed to COVID-19 infection. Since study enrolment commenced there has been a surge of COVID-19 infections in the states of Victoria and New South Wales, commencing in June 2021 and ongoing as of October 2021. This has resulted in a much more rapid vaccine rollout in these states and rapid enrolment into the SerOzNET study.

### Rationale for study

There remain significant unknowns regarding the response of cancer patients to the COVID-19 vaccine. In particular, in studies in Europe and the USA with relatively high background rates of COVID-19, the rollout of vaccination has been rapid. This has limited the ability to enrol large cohorts of cancer patients naïve to both COVID-19 infection and vaccination into studies of vaccine safety and immune response. Published studies to date have been in the context of rapid responses to unfolding events with pragmatic eligibility criteria; these studies have therefore included patients with heterogeneous clinical characteristics (related to both diagnosis and treatment) and have lacked the power to dissect contributing factors which might influence vaccine response such as treatment modality. Some have been unable to include a baseline blood sample due to rapid vaccine rollout, and others have had fragmented follow up due to repeated COVID-19 lockdowns and health service disruption.

Our study, SerOzNET, seeks to provide a comprehensive, prospective observational cohort of patients with baseline health status and blood tests collected, divided into balanced cohorts on different cancer therapies of interest. SerOzNET will enrol patients from a population with a very low rate of prior COVID-19 infection, and to date a low rate of COVID-19 vaccination. This will better inform clinicians in future regarding immune protection and safety of vaccines against COVID-19 in this vulnerable patient group.

## Methods

### Study design

The SerOzNET study objective is to characterise the response of patients with cancer, including children, to the currently available COVID-19 vaccinations in Australia. The aims of the study are to evaluate serologic and immunological responses to COVID-19 vaccination in predefined cohorts of patients with cancer in Australia, to evaluate short- and medium-term safety of COVID-19 vaccination in patients with cancer, and to evaluate the effect of COVID-19 vaccination on patient-reported quality of life.

SerOzNET is a multi-arm, multi-centre, prospective, observational cohort study of patients with cancer undergoing routine COVID-19 vaccination. Control samples will be provided by healthy controls enrolled on concurrent, separate studies with collaborating investigators, with blood tests taken at harmonised time points for comparison. Time points for sampling have also been harmonised with the publicly shared “SeroNet” protocol template released by the United States National Cancer Institute (NCI) to allow international comparisons [40]. To provide a nuanced understanding of outcomes depending on patient characteristics, this study will enrol patients falling into specific treatment cohorts and collect detailed demographic information.

Hypotheses:

1. The anamnestic humoral response to COVID-19 vaccine is likely to be blunted in cancer patients due to immunosuppression arising from their disease and/or treatment.
2. The T-lymphocyte-mediated vaccine response may also be modulated due to disease and treatment factors, with the impact of contemporary treatments such as immune checkpoint inhibitors of particular interest.
3. The adverse event profile may differ in cancer patients compared with the non-cancer population and therefore the impact of vaccine administration on quality of life of cancer patients is unknown.

The endpoints of SerOzNET relate to these hypotheses. The core primary endpoint is seroconversion rate following COVID-19 vaccine in each cohort compared with healthy controls at 1 month post second dose. Co-primary endpoints are translational research into immune correlates of COVID-19 vaccine in each of the cancer cohorts compared with healthy controls (qualitative and quantitative measures of humoral and cellular immunity) and assessment of patient-reported and medically determined adverse effects and quality of life following vaccination. Secondary endpoints include exploratory analysis of further immunologic parameters to characterise vaccine response and investigation of durability and kinetics of response in the months post vaccination.

### Setting

The SerOzNET lead study site is an outer-urban hospital in south-east Melbourne, Victoria with a large oncology department located over several campuses serving a culturally and linguistically diverse population. Patients will be approached during the course of their routine cancer care by a member of their care team (such as oncologist, haematologist, or nurse); further study advertisement will be via posters in waiting rooms and day treatment units which can facilitate self-referral. COVID-19 vaccination (standard of care, product administered dependent on current guidelines and availability) will occur at either a state-run site or local general practice according to availability and is not administered as part of the study itself: this approach was chosen so as to not complicate the running of the vaccine clinics or present confusion about responsibility for management of any acute toxicities. Study participants will attend the lead site at several timepoints relative to their COVID-19 vaccine for biospecimen collection. Surveys and scales for patient-reported quality of life and adverse events will be offered to patients preferably via email or SMS to complete remotely or via tablet while on-site, but for those without internet access paper surveys will be given.

Healthy controls are enrolled on parallel studies with collaborating investigators. Timepoints for blood collection and planned assays have been harmonised with the healthy control studies to allow direct comparison of outcome.

### Participants

Participants are current patients of our health service (or at collaborating cancer services). The cohorts to be enrolled are:

SOC or HM patients currently on cytotoxic chemotherapy.
SOC or HM patients currently on immunotherapy (immune checkpoint inhibitors).
(3a) SOC patients currently on systemic therapy which is not traditionally considered immunosuppressive (such as endocrine therapy, bone modifying agents or targeted therapy).
  (3b) Children with current or previous cancer treated only with radiotherapy, surgery, or surveillance.
Patients entering survivorship after having completed a course of cytotoxic chemotherapy between 6 and 12 months ago.
Patients with haematological malignancies characterised by hypogammaglobulinaemia or immunocompromise, such as multiple myeloma, CLL and low-grade lymphoma.
Patients with prior allergic reactions to PEG or polysorbate-80 containing compounds (this cohort will only be analysed for safety given their heterogeneous cancer types and treatments, unless concurrently enrolled in another cohort).

Up to 100 participants will be enrolled to each arm.

Inclusion criteria are deliberately broad to allow enrolment of large representative cohorts:

1. Age 12 years or older.
2. Cancer diagnosis and treatment fitting one of the above study cohorts.
3. Have not yet received a dose of COVID-19 vaccine, and eligible for vaccination as per current Australian government guidelines.

Exclusion criteria:

1. Unfit for serial blood collection (due to frailty, concurrent illness, poor venous access or other issue as determined by investigator).
2. Unable to consent, or parent or guardian unable to consent in the case of a patient < 18 years.
3. Estimated survival less than 12 months.

### Study activities

This study is observational and vaccine administration will be carried out through the usual channels (vaccine hubs, local health services) and not as part of the SerOzNET study itself. Participants will receive a standard-of-care COVID-19 vaccination supplied by the Australian government. The currently available products in Australia are the BNT162b2 (Pfizer) vaccine, and the ChAdOx1-S (Astra Zeneca) vaccine. The mRNA-1273 (Moderna) vaccine has recently become available and may also be incorporated into the study. As part of SerOzNET, administrative support is offered to participants to schedule and book their vaccination appointment when required.

#### Baseline characteristics

Detailed demographic information will be collected at baseline. Cancer diagnosis and treatment details will be recorded. The full list of common data elements to be collected is included in Additional file 1: Appendix S1.

#### Blood collection

Sample collection for serologic and immunologic analysis will be performed at timepoints shown in Table 2. Blood (45 mL for adults or participants > 50 kg body weight and 24 mL for children of body weight 50 kg or less) will be taken at baseline then at specified timepoints, with follow up on study for approximately 6 months, depending on vaccine product received. Blood will be collected and processed fresh into components (peripheral blood mononuclear cells (PBMC) and serum) and cryogenically stored for future batch-wise analysis, as outlined in Additional file 2: Appendix S2.

**Table 2:** SerOzNET blood sampling schedule

Week (W) and day (D) of trial	Pfizer 3-week interval	Astra Zeneca 6-week interval	Astra Zeneca 12-week interval	Moderna 4-week interval
*W0 D0 (− 7)	Baseline	Baseline	Baseline	Baseline
W3 D21 (± 3)	2nd dose		D21 post 1st dose	
W4 D28 (± 3)				2nd dose
W6 D42 (± 3)		2nd dose		
W7 D49 (± 1)	1 M post 2nd dose			
W8 D56				1 M post 2nd dose
W10 D70 (± 1)		1 M post 2nd dose		
W12 D84 (± 7)			2nd dose	
W15 D105 (± 7)	*3 M post 2nd dose			
W16 D112 (± 1)			1 M post 2nd dose	3 M post 2nd dose
W18 D126 (± 7)		3 M post 2nd dose		
W24 D168 (± 7)			3 M post 2nd dose	
W27 D189 (± 7)	6 M post 2nd dose			
W28 D196 (± 7)				6 M post 2nd dose

Additional vaccines can be added when available

*D* day, *W* week, *M* month

#### Collection of patient-reported outcomes and quality of life

Vaccine hesitancy will be collected at the time of enrolment using the Oxford COVID-19 Vaccine Confidence and Complacency Scale [41]. Quality of life will be assessed at multiple timepoints using the validated EORTC QLQ-C30 tool for adults, and the PedsQL measurement model for children [42, 43]. Patient-reported adverse effects will be collected 7 days after each vaccine dose using an online survey sent by email or text message, or paper survey comprising of Patient Reported Outcome- Common Terminology for Adverse Events (PRO-CTCAE) [44] items relevant to vaccine related adverse effects. Additional items of interest regarding delays in cancer treatment and hospital admissions will also be collected. The schedule is included in Table 3 and the full list of PROCTCAE items are listed in Additional file 3: Appendix S3.

**Table 3:** Patient reported outcomes and quality of life

	EORTC QLQC30 or PedsQL	Hesitancy scale	PRO CTCAE	Toxicity^a^
Vaccine dose 1 (− 7)	X	X		
D7 (± 1) post dose 1	X		X	
Vaccine dose 2	X			
D7 (± 1) post dose 2	X		X	
3 M post dose 2	X			X

*D* day, *M* month, *EORTC QLQ* European Organisation for Research and Treatment of Cancer Quality of Life Questionnaire, *PRO CTCAE* Patient Reported Outcomes Common Terminology Criteria for Adverse Events

^a^Medical record review

#### Toxicity

Apart from patient-reported outcomes, a medical review of records 3 months after the second dose of vaccine will be conducted to capture any serious adverse events as well as adverse events of interest (for example: delays in cancer treatment, blood clots, lymphadenopathy on imaging requiring further follow up). The database entry fields for this are presented in Additional file 1: Appendix S1.

### Analyses

Serum and PBMCs will be used for a number of core analyses. Participants will be invited to consent during the SerOzNET participant consent process to the storage of excess biospecimens after these core analyses are complete, for later exploratory analyses to be guided by preliminary results from our study and evolving understanding of COVID-19 immunity worldwide.

Core analyses will comprise qualitative and quantitative serologic assays and T-lymphocyte immune cell assessment. Serologic neutralisation assays against clinically relevant variants of COVID-19 and immunochemical testing will be performed to detect the presence and titre of anti-SARS-CoV-2 spike protein as previously published, which is correlated with early post-vaccine protection against symptomatic COVID-19 infection [45, 46]. Variants will be selected based on the vaccine (the variant that is identical to the Spike antigen used), the most immune evasive (presently this is Beta) and the dominant circulating variant globally (presently this is Delta). As aliquots of serum will be stored, any emerging variants of concern can be further tested for antibody breadth against vaccine convalescent serum controls courtesy of other vaccine and cohort studies that are continuing in Australia.

Immune cell assessment of T-lymphocyte function derived from PBMC fraction will comprise phenotypic analyses of peripheral T cell subsets and functional assays to measure cytokine production (e.g., interferon γ, method previously published [47]) and cytotoxic responses (e.g., CD107a expression) after co-culture with antigen derived from SARS-CoV-2 via flow cytometry. Controls for co-culture experiments will include mitogenic stimulus and peptides derived from common viruses (such as cytomegalovirus, Epstein–Barr virus, or influenza antigen in patients who have received influenza vaccine). Correlation of magnitude of serological response and T-cell assays will be described within study cohorts and between cohorts with healthy controls, to estimate impact of immunological aspects of disease and treatment on vaccine response. Recent data in immunocompromised patients on rheumatological disease modifying agents showed comparable immune cellular response to COVID-19 vaccine to controls, despite reduced rate of neutralising antibodies, suggesting potential heterogenous cellular and humoral immune response to vaccine in immunocompromised patients [48]. PBMCs will also be analysed for epigenetic markers of T cell dysfunction, effector status, and exhaustion.

### Sample size and statistical analysis

The sample size of 100 per cohort will have over 80% power (β of 0.80) to detect a decrease of 10% seroconversion rate in any cancer cohort (compared with assumed non-cancer population incidence of 95%) with 95% confidence (α of 0.05). T-lymphocyte correlates of vaccine response are less well-defined than the expected seroconversion rate, therefore this analysis has not been subject to formal statistical planning and comparison between groups via t-tests or analysis of variance will be for the purpose of hypothesis generation, with significant results deemed to be p < 0.05. Patient reported and medically reported toxicity will be analysed descriptively. Patient-reported quality of life will be analysed longitudinally.

### Ethics approval, trial registration and current status

This protocol was submitted to the Monash Health Human Research Ethics Committee (HREC) on 26 May 2021, and the amended final version (2.0) was approved on 22 June 2021. The trial was submitted for registration with the Australian New Zealand Clinical Trials Registry (registration number ACTRN12621001004853) in June 2021 prior to enrolment of first participant, with registration number granted on 30 July 2021. The first patient was enrolled on 25 June 2021. Recruitment is ongoing.

## Discussion

There are significant gaps in our understanding of immune response to COVID-19 vaccines in patients with different types and stages of cancer, and on various anti-cancer therapies, leaving uncertainty regarding both efficacy (including longevity of response) and safety. A further notable gap in the current literature relates to COVID-19 vaccine responses in paediatric cancer patients. This Australian study seeks to explore how adults and children with cancer respond to the COVID-19 vaccines both quantitatively (with in-depth assessment of immune correlates at clinically-relevant timepoints in relation to vaccine) and qualitatively through assessment of patient-reported measures. In addition, it also seeks to understand whether treatments generally thought not to affect the immune system (e.g., hormonal therapies) or to have had transient impact (e.g., chemotherapy completed 6 months prior to vaccination) might influence vaccine efficacy. Furthermore, the study will link patient and physician reported toxicity and quality of life data. All of the findings are likely to influence clinical practice regarding timing and potentially type of vaccinations and booster doses in future.

We also seek to provide more detailed safety information regarding COVID-19 vaccination in this vulnerable group, to better inform the cancer patient population and hopefully provide reassurance to those with vaccine hesitancy. Our Australian population has had limited COVID-19 exposure in the community which has led to a perceived lack of risk of future COVID-19 infection, and subsequent reduced acceptance of potential vaccine side effects. More detailed information regarding safety in the cancer population could help address patient anxiety about vaccination while on cancer therapy.

An unintended benefit of our study to date has been the ability to facilitate COVID-19 vaccination for our vulnerable cohort, through both access to oncologists in the study team to provide counsel regarding vaccination, and practical assistance with vaccination bookings. We will further explore the rate of participants requiring assistance as we continue to recruit and publish this data to inform service provision in future.

Overall, we seek to add to the understanding of COVID-19 vaccination for our patient group, to better inform physicians and patients regarding preventive health care during the ongoing pandemic.

## Supplementary Information

**Additional file 1: Appendix S1.** Common data element collection for SerOzNET.

**Additional file 2: Appendix S2.** Biospecimen handling.

**Additional file 3: Appendix S3.** SerOzNET Patient Reported Outcomes Common Terminology Criteria for Adverse Events: PRO-CTCAE.

## Data Availability

The datasets generated during the current study are not publicly available due to ongoing patient enrolment and data collection. The data fields being collected are available in Additional file 1: Appendix S1 of this paper. The dataset generated during the current study will be available from the corresponding author on reasonable request at study completion.

## Abbreviations

ANZCTR: Australia New Zealand Clinical Trials Registry
COVID-19: Coronavirus disease 2019
ESMO: European Society of Medical Oncology
HM: Haematological malignancy
iRAE: Immune related adverse effects
OECD: Organisation for Economic Cooperation and Development
PBMC: Peripheral blood mononuclear cells
PEG: Polyethylene glycol
SOC: Solid organ cancer
SOT: Solid organ transplant
