# Supplementary material for: Protocol for SARS-CoV-2 post-vaccine surveillance study in Australian adults and children with cancer: an observational study of safety and serological and immunological response to SARS-CoV-2 vaccination (SerOzNET)
Source: BMC Infect Dis. 2022 Jan 20;22:70. doi: 10.1186/s12879-021-07019-1 (PMC8771167; doi:10.1186/s12879-021-07019-1)
Supplement: Supplementary file 1 — Additional file 1: Appendix S1. Common data element collection for SerOzNET. [file 12879_2021_7019_MOESM1_ESM.docx]

Appendix S1 Common data element collection for SerOzNET

Some elements in the table are adapted from the publicly available “SeroNet” protocol^1^

**Registration**

| **CDE Name** | **CDE Mapping Specification** | **Response Source** | **Measure Source** | **Definition** |
| --- | --- | --- | --- | --- |
| **Unique ID** |  | Calculated from site of enrolment and registration number |  | Unique identification number comprised of auto generated enrolment number by electronic database and site of enrolment |
| **Age*** | Collected  or  Calculated from Birth Date MM/DD/YYYY | Medical record | Age calculated from date of birth | Years since birth |
| **Sex*** | Male\| Female\| None of these describe me (Optional free text)^\| Prefer not to answer\| Unknown | Self-Report | Patient | The physical sexual characteristics of the neonate at birth. (NCI Thesaurus Concept Code: C124436)     *Question text/instruction: What was your biological sex assigned at birth?* |
| **Ethnicity*** | 1 OCEANIAN 2 NORTH-WEST EUROPEAN 3 SOUTHERN AND EASTERN EUROPEAN 4 NORTH AFRICAN AND MIDDLE EASTERN 5 SOUTH-EAST ASIAN 6 NORTH-EAST ASIAN 7 SOUTHERN AND CENTRAL ASIAN 8 PEOPLES OF THE AMERICAS 9 SUB-SAHARAN AFRICAN | Self-Report | 1249.0 Australian Standard Classification of Cultural and Ethnic Groups, 2019 available from https://www.abs.gov.au/statistics/classifications/australian-standard-classification-cultural-and-ethnic-groups-ascceg/latest-release#data-downloads | A geographic ancestral origin category that is assigned to a population group based mainly on physical characteristics that are thought to be distinct and inherent. (NCI Thesaurus Concept Code: C17049)    *Question text/Instruction: What is your race or ethnicity?* |
| **Aboriginal or Torres Strait Islander*** | Aboriginal\| Torres Strait Islander\|Both Aboriginal and Torres Strait Islander \|Not Aboriginal or Torres Strait Islander Unknown | Self-Report |  | Question text/Instruction: Do you identify as Aboriginal or Torres Strait Islander |
| **Country of birth** |  | Self report |  |  |
| **Language spoken** |  | Self report |  | Language spoken at home- "What language do you speak at home with your family?” |
| **Performance Status Assessment Eastern Cooperative Oncology Group Scale (ECOG)** | 0\| 1\|2\| 3\| 4\| 5 | Medical record as per last medical oncologist review before enrolment | Eastern Co- operative Oncology Group performance status^2^ | *The ECOG functional performance status of the patient/participant.  0 Fully active, able to carry on all pre-disease performance without restriction. 1 Restricted in physically strenuous activity but ambulatory and able to carry out work of a light or sedentary nature, e.g., light housework, office work. 2 Ambulatory and capable of all selfcare but unable to carry out any work activities. Up and about more than 50% of waking hours. 3 Capable of only limited selfcare, confined to bed or chair more than 50% of waking hours. 4 Completely disabled. Cannot carry on any selfcare. Totally confined to bed or chair. 5 Dead* |
| **Smoking** | Current smoker Former smoker  Never smoker | Self-report |  |  |
| **Informed Consent Signed** | Yes\| No\| |  |  | *Question text/Instructions: Was informed consent signed?* |
| **Informed consent signed date** | MM/DD/YYYY\| N/A or not reported | Abstracted | Informed consent form | *Question text/Instructions: When was informed consent signed?* |
| **Consent type** | Written informed consent by the participant/subject | Abstracted | Informed consent form | *Question text/Instructions: What type of informed consent was used?* |
| **COVID-19 Positive Test Result previously** | Yes\| No\| Pending\|  Unknown | Self report | Self report from patient | Have you ever had a positive COVID19 test/ been diagnosed with COVID19/Coronavirus infection |
| **Date of Diagnosis Test Result** | DDMMYYYY | Medical record or self report if record not available |  | *Question text/instruction: When were the test results received*? |
| **Influenza vaccination in 2021 prior to study commencement** | Yes \| No \| Unknown \|Prefer not to answer | Self report |  |  |
| **Influenza vaccination date** | DDMMYYYY | Self report |  |  |

**Vaccination details**

| **CDE Name** | **CDE Response** | **Response Source** | **Definition** |
| --- | --- | --- | --- |
| **COVID-19 Vaccine Manufacturer** | Pfizer \| Moderna \| AstraZeneca \| Unknown \| Other (specify) | Booking details | A textual description of the pharmaceutical manufacturer of COVID-19 vaccine. |
| **COVID-19 Vaccination Date – 1^st^ dose** | MMDDYYYY | Booking details | The response indicating the date the initial COVID-19 vaccine immunization was administered. |
| **COVID-19 Vaccination Date – 2^nd^ dose** | MMDDYYYY | Booking details | The response indicating the date the booster COVID-19 vaccine immunization was administered. |
| **Influenza vaccination in 2021 after study commencement** | Yes \| No \| Unknown \|Prefer not to answer | Self report |  |
| **Influenza vaccination date after study entry** | MMDDYYYY |  |  |

**Cancer diagnosis details**

| **CDE Name** | **CDE Response** | **Response Source** | **Definition** |
| --- | --- | --- | --- |
| **Disease/Condition type** | See list- ICD-10, can also be free text if not included in ICD-10 | Medical record | Disease or Disorder Present Name (see table below ICD-10) with a focus on cancer and autoimmune diseases |
| **Cancer stage** | Stage I\|  Stage II\|  Stage III\|  Stage IV\|  Other not included in AJCC (free text)\| | Medical record | As per AJCC 8 classification |
| **Treatment intent** | Palliative  Curative  Neoadjuvant  Adjuvant  Supportive | Cancer treatment plan |  |
| **Cancer Subtype** | Histologic subtype eg. Adenocarcinoma, sarcoma etc | Medical record | Disease or Disorder Present Histologic Subtype Text |
| **Cancer treatment type** | Chemotherapy (current)\| Immunotherapy (current)\| Hormone therapy\| Bone modifying agent\| Other targeted therapy \|Chemotherapy (previous within 12 months) | Medical record | Regimen Administered |
| **Therapy Regimen Ongoing/Ceased** | Ongoing\| Ceased | Medical record | Last Treatment Administered Date |
| **If ceased- date** | DDMMYY | Pharmacy Chemotherapy Summary |  |
| **Therapy Regimen Name** | fill-in | Pharmacy Chemotherapy Summary | Therapeutic Procedure Regimen Since Last Treatment Administered Name |
| **Date of most recent dose of therapy prior to vaccine dose 1** | DDMMYY | Medical record |  |
| **Steroid use** | Yes on chemo day only  Yes day of chemo and for 1-3 days after chemo  Yes treatment dose daily (e.g. brain mets, liver capsule pain, treatment irAE)  Yes replacement dose only (e.g. hydrocortisone for adrenal insufficiency)  No | Medical record |  |
| **Steroid name** | Dexamethasone  Prednisolone  Hydrocortisone  Other | Medical record |  |
| **Steroid dose prednisolone equivalent in mg/day** |  | Medical record | <https://www>.mdcalc.com/steroid-conversion-calculato r 1.5mg dexamethasone =10mg prednisolone. 40mg hydrocortisone= 10mg prednisolone. 8mg methylprednisolone =10mg prednisolone |
| **If not currently on chemotherapy, has the patient ever received chemotherapy** | Y/N  Date ceased | Medical record |  |

**Comorbidities**

| **CDE Name** | **CDE Response** | **Response Source** | **Definition** |
| --- | --- | --- | --- |
| **Comorbidities*** | Alcoholism\|  Diabetes type I\|  Diabetes type II\|  Obesity\|  Chronic kidney disease\|  Dementia\|  Alzheimer’s\|  Chronic liver disease\|  Other Chronic respiratory disease (e.g.COPD, Emphysema)\|  Asthma\|  Chronic oxygen requirement\| Coronary artery disease\|  Epilepsy\|  Multiple sclerosis\|  Other Chronic Neurological condition (**specify)**\| Hepatitis\|  Hypertension\|  Congestive heart failure\|  Cancer\|  Immune deficiency/HIV/AIDS\|  Autoimmune/Immuno compromised condition\|  Psychological/psychiatric condition\|  Substance Use Disorders\|  Other chronic diseases (**specify**)  For each item: Yes/No/Unknown/N/A or not reported | Medical record | Any abnormal condition of the body or mind that causes discomfort, dysfunction, or distress to the person affected or those in contact with the person. The term is often used broadly to include injuries, disabilities, syndromes, symptoms, deviant behaviours, and atypical variations of structure and function.(NCI Thesaurus Concept Code: C2991) Condition is a state of being, such as a state of health. (NCI Thesaurus Concept Code: C25457)   Concurrent Disease Type: The classification and naming of comorbid conditions. (NCI Thesaurus Concept Code: C164326)  *Question text/instructions:  Significant Underlying medical conditions at the time of COVID-19 vaccination* |
| **Previous blood clot** | Yes- previous DVT or PE Yes- previous arterial thromboembolism  Yes- other venous clot  No | Medical record |  |
| **Charlson comorbidity score** | Age  Myocardial infarction  Chronic heart failure  Peripheral vascular disease  CVA or TIA  Dementia  COPD  Connective tissue disease  Peptic ulcer disease  Liver disease  Diabetes mellitus  Hemiplegia  Moderate to severe CKD  Solid tumor (other than the cancer under study)  Leukemia  Lymphoma  AIDS | Medical record | Score as per Charlson comorbidity index scoring^3^ |

**Toxicity**

| **CDE Name** | **CDE Response** | **Response Source** | **Measure Source** | **Definition** |
| --- | --- | --- | --- | --- |
| **Serious adverse event during study period** | None\| If yes- code as per CTCAE | Medical record | CTCAE v 5.0 | <https://ctep.cancer.gov/protocoldevelopment/electronic_applications/docs/CTCAE_v5_Quick_Reference_5x7.pdf> |
| **SAE likely attributable to vaccine** | Yes- definitely or very likely  Possibly- equally likely as another cause  Unlikely- another cause identified (as per treating physician opinion) | As per treating physician opinion | Medical record |  |
| **Cancer treatment delay during study period** | Yes/No |  | Pharmacy chemotherapy summary or clinical notes |  |
| **Cancer treatment delay during study period due to vaccine related AE** | Yes/No/unclear | As per treating physician opinion | Medical record |  |
| **Cancer treatment modification during study period** | Yes- completed planned/ Yes- intolerance/ Yes- disease progression/ Yes – other insert reason/ No | Clinical notes | Medical record |  |
| **Cancer treatment modification due to vaccine related AE** | Yes/No | As per treating physician opinion | Medical record |  |
| **Thrombotic events** | Yes- deep vein thrombosis/ Yes – pulmonary embolism/ Yes- other venous thrombosis/ Yes – coronary artery event/ Yes- cerebrovascular event/ Yes- other arterial thrombus (state)/ No | Clinic review note, discharge summary, letter or discharge summary from outside provider | Medical record |  |
| **Allergic reaction** | Yes- Type I hypersensitivity/ Yes- Type II hypersensitivity/ Yes- Type III hypersensitivity/ Yes-Type IV hypersensitivity/ Yes- unknown type (describe in comment)/ No |  |  |  |
| **Decline in performance status** | Yes/No | Medical record ECOG determination | Medical record | Compared to ECOG at enrolment |
| **Other adverse event likely attributable to vaccine** | Yes/No | As per treating physician opinion | Medical record | Type of AE- classify by CTCAE v 5.0 with event term and grade |
| **Lymphadenopathy detected on imaging during study period** | Yes/No | As per radiology report | Medical record/ radiology reports |  |
| **Lymphadenopathy detected on imaging during study period potentially due to vaccination** | Yes/No/Unknown | As per treating physician opinion | Medical record/ radiology reports |  |
| **Additional investigation required due to lymphadenopathy** | Yes- early reimaging  Yes- different imaging modality  Yes- biopsy  No |  | Medical record/ radiology reports |  |
| **Lymphadenopathy attributed to vaccine after investigation?** | Yes/No | As per treating physician opinion | Medical record/ radiology reports |  |

**ICD-10 diagnosis codes**^4^

| Neoplasms (C00–D48) |
| --- |
| Malignant neoplasms of lip, oral cavity and pharynx (C00-C14) |
|  |
| Malignant neoplasm of lip (C00) |
| Malignant neoplasm of base of tongue (C01) |
| Malignant neoplasm of other and unspecified parts of tongue (C02) |
| Malignant neoplasm of gum (C03) |
| Malignant neoplasm of floor of mouth (C04) |
| Malignant neoplasm of palate (C05) |
| Malignant neoplasm of other and unspecified parts of mouth (C06) |
| Malignant neoplasm of parotid gland (C07) |
| Malignant neoplasm of other and unsp major salivary glands (C08) |
| Malignant neoplasm of tonsil (C09) |
| Malignant neoplasm of oropharynx (C10) |
| Malignant neoplasm of nasopharynx (C11) |
| Malignant neoplasm of pyriform sinus (C12) |
| Malignant neoplasm of hypopharynx (C13) |
| Malig neoplasm of sites in the lip, oral cavity and pharynx (C14) |
| Malignant neoplasms of digestive organs (C15-C26) |
|  |
| Malignant neoplasm of esophagus (C15) |
| Malignant neoplasm of stomach (C16) |
| Malignant neoplasm of small intestine (C17) |
| Malignant neoplasm of colon (C18) |
| Malignant neoplasm of rectosigmoid junction (C19) |
| Malignant neoplasm of rectum (C20) |
| Malignant neoplasm of anus and anal canal (C21) |
| Malignant neoplasm of liver and intrahepatic bile ducts (C22) |
| Malignant neoplasm of gallbladder (C23) |
| Malignant neoplasm of other and unsp parts of biliary tract (C24) |
| Malignant neoplasm of pancreas (C25) |
| Malignant neoplasm of other and ill-defined digestive organs (C26) |
| Malignant neoplasms of respiratory and intrathoracic organs (C30-C39) |
|  |
| Malignant neoplasm of nasal cavity and middle ear (C30) |
| Malignant neoplasm of accessory sinuses (C31) |
| Malignant neoplasm of larynx (C32) |
| Malignant neoplasm of trachea (C33) |
| Malignant neoplasm of bronchus and lung (C34) |
| Malignant neoplasm of thymus (C37) |
| Malignant neoplasm of heart, mediastinum and pleura (C38) |
| Malig neoplm of sites in the resp sys and intrathorac organs (C39) |
| Malignant neoplasms of bone and articular cartilage (C40-C41) |
|  |
| Malignant neoplasm of bone and articular cartilage of limbs (C40) |
| Malignant neoplasm of bone/artic cartl of and unsp sites (C41) |
| Melanoma and other malignant neoplasms of skin (C43-C44) |
|  |
| Malignant melanoma of skin (C43) |
| Other and unspecified malignant neoplasm of skin (C44) |
| Merkel cell carcinoma (C4A) |
| Malignant neoplasms of mesothelial and soft tissue (C45-C49) |
|  |
| Mesothelioma (C45) |
| Kaposi's sarcoma (C46) |
| Malignant neoplasm of prph nerves and autonomic nervous sys (C47) |
| Malignant neoplasm of retroperitoneum and peritoneum (C48) |
| Malignant neoplasm of other connective and soft tissue (C49) |
| Malignant neoplasms of breast (C50) |
|  |
| Malignant neoplasm of breast (C50) |
| Malignant neoplasms of female genital organs (C51-C58) |
|  |
| Malignant neoplasm of vulva (C51) |
| Malignant neoplasm of vagina (C52) |
| Malignant neoplasm of cervix uteri (C53) |
| Malignant neoplasm of corpus uteri (C54) |
| Malignant neoplasm of uterus, part unspecified (C55) |
| Malignant neoplasm of ovary (C56) |
| Malignant neoplasm of other and unsp female genital organs (C57) |
| Malignant neoplasm of placenta (C58) |
| Malignant neoplasms of male genital organs (C60-C63) |
|  |
| Malignant neoplasm of penis (C60) |
| Malignant neoplasm of prostate (C61) |
| Malignant neoplasm of testis (C62) |
| Malignant neoplasm of other and unsp male genital organs (C63) |
| Malignant neoplasms of urinary tract (C64-C68) |
|  |
| Malignant neoplasm of kidney, except renal pelvis (C64) |
| Malignant neoplasm of renal pelvis (C65) |
| Malignant neoplasm of ureter (C66) |
| Malignant neoplasm of bladder (C67) |
| Malignant neoplasm of other and unspecified urinary organs (C68) |
| Malignant neoplasms of eye, brain and other parts of central nervous system (C69-C72) |
|  |
| Malignant neoplasm of eye and adnexa (C69) |
| Malignant neoplasm of meninges (C70) |
| Malignant neoplasm of brain (C71) |
| Malig neoplm of spinal cord, cranial nerves and oth prt cnsl (C72) |
| Malignant neoplasms of thyroid and other endocrine glands (C73-C75) |
|  |
| Malignant neoplasm of thyroid gland (C73) |
| Malignant neoplasm of adrenal gland (C74) |
| Malignant neoplasm of endo glands and related structures (C75) |
| Malignant neuroendocrine tumors (C7A) |
|  |
| Malignant neuroendocrine tumors (C7A) |
| Secondary neuroendocrine tumors (C7B) |
|  |
| Secondary neuroendocrine tumors (C7B) |
| Malignant neoplasms of ill-defined, other secondary and unspecified sites (C76-C80) |
|  |
| Malignant neoplasm of other and ill-defined sites (C76) |
| Secondary and unspecified malignant neoplasm of lymph nodes (C77) |
| Secondary malignant neoplasm of resp and digestive organs (C78) |
| Secondary malignant neoplasm of other and unspecified sites (C79) |
| Malignant neoplasm without specification of site (C80) |
| Malignant neoplasms of lymphoid, hematopoietic and related tissue (C81-C96) |
|  |
| Hodgkin lymphoma (C81) |
| Follicular lymphoma (C82) |
| Non-follicular lymphoma (C83) |
| Mature T/NK-cell lymphomas (C84) |
| Other and unspecified types of non-Hodgkin lymphoma (C85) |
| Other specified types of T/NK-cell lymphoma (C86) |
| Malignant immunoproliferative disease and certain other B-cell lymph (C88) |
| Multiple myeloma and malignant plasma cell neoplasms (C90) |
| Lymphoid leukemia (C91) |
| Myeloid leukemia (C92) |
| Monocytic leukemia (C93) |
| Other leukemias of specified cell type (C94) |
| Leukemia of unspecified cell type (C95) |
| Other & unspecified malignant neoplasm of lymphoid, hematopoietc and rel tiss (C96) |
| In situ neoplasms (D00-D09) |
|  |
| Carcinoma in situ of oral cavity, esophagus and stomach (D00) |
| Carcinoma in situ of other and unspecified digestive organs (D01) |
| Carcinoma in situ of middle ear and respiratory system (D02) |
| Melanoma in situ (D03) |
| Carcinoma in situ of skin (D04) |
| Carcinoma in situ of breast (D05) |
| Carcinoma in situ of cervix uteri (D06) |
| Carcinoma in situ of other and unspecified genital organs (D07) |
| Carcinoma in situ of other and unspecified sites (D09) |
| Benign neoplasms, except benign neuroendocrine tumors (D10-D36) |
|  |
| Benign neoplasm of mouth and pharynx (D10) |
| Benign neoplasm of major salivary glands (D11) |
| Benign neoplasm of colon, rectum, anus and anal canal (D12) |
| Benign neoplasm of and ill-defined parts of digestive system (D13) |
| Benign neoplasm of middle ear and respiratory system (D14) |
| Benign neoplasm of other and unsp intrathoracic organs (D15) |
| Benign neoplasm of bone and articular cartilage (D16) |
| Benign lipomatous neoplasm (D17) |
| Hemangioma and lymphangioma, any site (D18) |
| Benign neoplasm of mesothelial tissue (D19) |
| Benign neoplm of soft tissue of retroperiton and peritoneum (D20) |
| Other benign neoplasms of connective and other soft tissue (D21) |
| Melanocytic nevi (D22) |
| Other benign neoplasms of skin (D23) |
| Benign neoplasm of breast (D24) |
| Leiomyoma of uterus (D25) |
| Other benign neoplasms of uterus (D26) |
| Benign neoplasm of ovary (D27) |
| Benign neoplasm of other and unspecified female genital organs (D28) |
| Benign neoplasm of male genital organs (D29) |
| Benign neoplasm of urinary organs (D30) |
| Benign neoplasm of eye and adnexa (D31) |
| Benign neoplasm of meninges (D32) |
| Benign neoplasm of brain and other parts of central nervous system (D33) |
| Benign neoplasm of thyroid gland (D34) |
| Benign neoplasm of other and unspecified endocrine glands (D35) |
| Benign neoplasm of other and unspecified sites (D36) |
| Benign neuroendocrine tumors (D3A) |
|  |
| Benign neuroendocrine tumors (D3A) |
| Neoplasms of uncertain behaviour, polycythemia vera and myelodysplastic syndromes (D37-D48) |
|  |
| Neoplasm of uncertain behaviour of oral cavity and digestive organs (D37) |
| Neoplasm of uncertain behavior of mid ear & resp and intrathoracic org (D38) |
| Neoplasm of uncertain behaviour of female genital organs (D39) |
| Neoplasm of uncertain behaviour of male genital organs (D40) |
| Neoplasm of uncertain behaviour of urinary organs (D41) |
| Neoplasm of uncertain behaviour of meninges (D42) |
| Neoplasm of uncertain behaviour of brain and central nervous system (D43) |
| Neoplasm of uncertain behaviour of endocrine glands (D44) |
| Polycythemia vera (D45) |
| Myelodysplastic syndromes (D46) |
| Other neoplasm of uncertain behaviour of lymphoid, hematopoietic & related tissue (D47) |
| Neoplasm of uncertain behaviour of other and unspecified sites (D48) |
| Neoplasms of unspecified behaviour (D49) |
|  |
| Neoplasms of unspecified behaviour (D49) |

1. SeroNet Resources for Researchers and Clinicians | Center for Strategic Scientific Initiatives (CSSI) [Internet]. [cited 2021 Jul 6]. Available from: https://cssi.cancer.gov/seronet

2. Oken MM, Creech RH, Tormey DC, Horton J, Davis TE, McFadden ET, et al. Toxicity and response criteria of the Eastern Cooperative Oncology Group. American journal of clinical oncology. 1982 Dec;5(6):649–55.

3. Charlson M, Pompei P, Ales K, MacKenzie C. A new method of classifying prognostic comorbidity in longitudinal studies: development and validation. Journal of chronic diseases [Internet]. 1987 [cited 2021 Aug 4];40(5):373–83. Available from: https://pubmed.ncbi.nlm.nih.gov/3558716/

4. 2021 ICD-10-CM | CMS [Internet]. [cited 2021 Aug 4]. Available from: https://www.cms.gov/medicare/icd-10/2021-icd-10-cm
