# Supplementary material for: Protocol for SARS-CoV-2 post-vaccine surveillance study in Australian adults and children with cancer: an observational study of safety and serological and immunological response to SARS-CoV-2 vaccination (SerOzNET)
Source: BMC Infect Dis. 2022 Jan 20;22:70. doi: 10.1186/s12879-021-07019-1 (PMC8771167; doi:10.1186/s12879-021-07019-1)
Supplement: Supplementary file 2 — Additional file 2: Appendix S2. Biospecimen handling. [file 12879_2021_7019_MOESM2_ESM.docx]

Appendix S2

Biospecimen handling

1. Serum

(adapted from BD Vacutainer product information^1^ and NCI SeroNet Serum Biospecimen Processing Procedure^2^)

- 1. Venepuncture performed, blood is collected into an 8.5mL BD Vacutainer SST tube
  2. Tube inverted gently no more than 8 times
  3. Blood is allowed to clot for 30-60 minutes
  4. Tube containing whole blood is centrifuged for 12 minutes at 1200g within one hour of collection
  5. Serum is collected with a sterile pipette and transferred to labelled sterile 5mL storage vials and transferred to -80 °C freezer.

1. Peripheral blood mononuclear cells (adapted from SepMate product information^3^)

ACD (anticoagulant Tri-sodium Citrate with Citric Acid and Dextrose) Vacutainer tube 8.5mL: 4 tubes

Total volume of: 34ml

Pool blood samples from a single patient into 1 x 50mL tube then dilute 1:1 with sterile 2% phosphate buffered solution (PBS) and 2% fetal calf serum (FCS).

Add 15mls of density gradient medium into each SepMate tube by carefully pipetting it through the central hole of the SepMate insert.

Keeping the SepMate tube vertical, the diluted sample is carefully added by pipetting it slowly and gently down the side of the tube, taking care not to let the sample run through the central hole of the tube. Maximum of 17.5 mL of diluted blood per SepMate tube.

Centrifuge tubes at 1200g for 12mins with brake on at room temperature.

Decant the top layer, which contains the enriched PBMCs, in one swift motion into a prepared 50mL tube.

Top up each tube to 50mL with 2% FBS/PBS and centrifuge at 300g for 8 mins with brake on at room temperature.

Decant supernatant and resuspend cells in 1mL 2% PBS/FBS and merge both tubes into one tube. Then top up the tube to 10mL with 2% FBS/PBS. Remove 100uL for cell counting with Trypan Blue.

Centrifuge tubes at 300g for 8 mins with brake on at room temperature. Decant supernatant, resuspend cells in 2%PBS/FBS and put on ice.

Freeze down cell pellets in freeze media (90%FCS 10%DMSO) and then aliquot in cryovials (5x10^6^ cells/vial) and freeze down at -80.

1. Becton Dickinson and Company. Vacutainer® Evacuated Blood Collection System [Internet]. 2018. Available from: file:///C:/Users/amybo/Downloads/PAS_BC_Vacutainer-Blood-Collection-In-Vitro-System_DF_EN.pdf

2. Kemp T. Serum Biospecimen Processing Procedure (NCI SeroNet Guidance Document) [Internet]. Frederick National Laboratory for Cancer Rsearch Vaccine, Immunity and Cancer Program Standard Operating Procedure. 2020 [cited 2021 Aug 11]. Available from: https://cssi.cancer.gov/sites/default/files/seronet-study-template-vaccine-studies-v2.pdf

3. SepMate^TM^ PBMC Isolation Tubes [Internet]. [cited 2021 Aug 11]. Available from: https://www.stemcell.com/products/brands/sepmate-pbmc-isolation.html
