# Supplementary material for: Protocol for SARS-CoV-2 post-vaccine surveillance study in Australian adults and children with cancer: an observational study of safety and serological and immunological response to SARS-CoV-2 vaccination (SerOzNET)
Source: BMC Infect Dis. 2022 Jan 20;22:70. doi: 10.1186/s12879-021-07019-1 (PMC8771167; doi:10.1186/s12879-021-07019-1)
Supplement: Supplementary file 3 — Additional file 3: Appendix S3. SerOzNET Patient Reported Outcomes Common Terminology Criteria for Adverse Events: PRO-CTCAE. [file 12879_2021_7019_MOESM3_ESM.docx]

**Appendix S3.**

**SerOzNET Patient Reported Outcomes Common Terminology Criteria**

**for Adverse Events: PRO-CTCAE**

Solicited adverse reaction list based on the COV001 trial (Astra Zeneca, NCT04324606) and the BNT162b2 study (Pfizer,

NCT04368728), plus investigator-added additional specific points of interest relating to serious adverse events and cancer

treatment. Common data elements from the PRO-CTCAE as recommended in the NCI SeroNET protocol where applicable.

**1. Did you have pain at the injection site?**

 No

 Mild- did not interfere with doing my usual activities

 Moderate- slowed me down or made my usual activities more difficult

 Severe- stopped me doing my usual activities

 Serious- I needed to attend the emergency department due to pain at the

injection site

**2. Did you have redness or swelling at the injection site?**

 No

 Minor- 2 to 5cm area of redness or swelling

 Moderate- 5 to 10cm area of redness or swelling

 Severe- more than 10cm (most of upper arm affected)

 Serious- blistering, skin damage or soft tissue damage requiring medical attention

**3. Did you have itch at the injection site?**

 No

 None

 Mild

 Moderate

 Severe

 Very severe

**4. Have you had a fever after the vaccination?**

 No

 Yes- I felt feverish but did not take temperature

 Yes- mild 37.5-37.9 degrees

 Yes- moderate 38-38.5 degrees

 Yes- high fever 38.6 degrees or higher

 Yes- went to hospital for assessment or treatment

**5. Did you take any medication to treat side effects after your vaccine?**

 Yes- paracetamol

 Yes – anti-inflammatory for example ibuprofen (Nurofen), diclofenac (Voltaren),

naproxen (Naprosyn)

 Yes- opioid pain killer – oxycodone (Endone), codeine (Panadeine or Panadeine

Forte)

 Yes- antihistamine

 Yes- other- specify ________________________________

**6. Did you have new or worsened fatigue since your vaccination? YES / NO** (please

circle)

***6a. If yes****, in the last 7 days, how much did fatigue, tiredness, or lack of energy*

*interfere with your usual or daily activities?*

 Not at all

 A little bit

 Somewhat

 Quite a bit

 Very much

**7. Did you have new or worsened headache after your vaccination? YES / NO** (please

circle)

***7a. If yes****, in the last 7 days, what was the severity of your headache at its worst?*

 None

 Mild

 Moderate

 Severe

 Very severe

**8. In the last 7 days, how often did you have shivering or shaking chills?**

 Almost constantly

 Frequently

 Occasionally

 Rarely

 Never

**9. Did you have new or worsened muscle pain since your vaccine dose? YES / NO**

(please circle)

***9a. If yes, in the last 7 days, what was the severity of your aching muscles at their worst?***

 None

 Mild

 Moderate

 Severe

 Very severe

**10. Did you have new or worsened joint pain since your vaccination? YES / NO** (please

circle)

***10a. If yes,*** *in the last 7 days, how much did aching joints (such as elbows, knees,*

*shoulders) interfere with your usual or daily activities?*

 Not at all

 A little bit

 Somewhat

 Quite a bit

 Very much

**11. Did you have new or worsened nausea since your vaccination? YES / NO** (please

circle)

***11a. If yes,*** *in the last 7 days, how often did you have nausea?*

 Almost constantly

 Frequently

 Occasionally

 Rarely

 Never

***11b. If yes,*** *in the last 7 days, what was the severity of your nausea at its worst?*

 None

 Mild

 Moderate

 Severe

 Very severe

**12. Did you have new or worsened vomiting since your vaccination? YES / NO** (please

circle)

4

**12a. In the last 7 days, what was the severity of your vomiting at its worst?**

 Almost constantly

 Frequently

 Occasionally

 Rarely

 Never

**13. Did you have new or worsened diarrhoea since your vaccination? YES / NO** (please

circle)

**13a. If yes, in the last 7 days, how often did you have loose or watery stools**

**(diarrhoea)?**

 Almost constantly

 Frequently

 Occasionally

 Rarely

 Never

**13b. If yes, how severe was your diarrhoea?**

**No diarrhoea / Mild / Moderate / Severe** (please circle one)

*[****Mild:*** *2 to 3 loose stools in 24 hours;* ***Moderate:*** *4 to 5 loose stools in 24 hours; or* ***Severe:*** *6*

*or more loose stools in 24 hours]*

**14. In the last 7 days, did you have any RASH? YES / NO** (please circle)

**15. In the last 7 days, did you experience HIVES (itchy, red bumps on the skin)? YES /**

**NO** (please circle)

**16. In the last 7 days, what was the severity of your wheezing (whistling noise in the**

**chest when breathing) at its worst?**

 None

 Mild

 Moderate

 Severe

 Very severe

**17. Have you had to delay your anticancer treatment in the last 7 days (since**

**vaccination)? YES / NO** (please circle)

**18. Have you attended the emergency department in the last 7 days (since**

**vaccination)?**

 No

 Yes (Monash Health: Clayton, Dandenong or Casey Emergency Dept.)

 Yes (other site- please specify where ____________________________________ )

**19. Have you been admitted to hospital for any reasons in the last 7 days (since**

**vaccination)?**

 No

 Yes (Monash Health: Clayton, Dandenong , Casey, Moorabbin, Kingston)

 Yes (other site- please specify where _________________________________ )

**20. Have you started new treatment for a blood clot in the last 7 days (since**

**vaccination)? YES / NO** (please circle)

**21. Have you needed to see your local doctor in the last 7 days (since vaccination)?**

 No

 Yes- routine check or prescription

 Yes – unwell (Please specify the issue _________________________________)
